# Imaginaries of Invention Management: Comparing Path Dependencies in East and West Germany

**DOI:** 10.1007/s11024-018-9347-3

**Published:** 2018-02-13

**Authors:** Lisa Sigl, Liudvika Leišytė

**Affiliations:** 1 Center for Higher Education (zhb), Faculty of Business and Economics, TU Dortmund University, Vogelpothsweg 78, 44227 Dortmund, Germany; 2 Research Platform Responsible Research and Innovation in Academic Practice, University of Vienna, Universitätsstrasse 7, 1010 Vienna, Austria

**Keywords:** invention management, patents, sociotechnical imaginaries, path dependency, technology transfer, Germany, comparison

## Abstract

The ways in which societies and institutions institutionalize and practice invention management reflects not only how new ideas are valued, but also imaginaries about the role of science and technology for societal development. Often taking the US Bayh-Dole-Act as a model, many European states have recently implemented changes in how inventions at academic institutions are to be handled to optimize their societal impact. We analyze how these changes have been taken up—and made sense of—in regions with different pre-existing infrastructures, practices and semantics of invention management. For doing so, we build on a comparative analysis of continuities and changes in infrastructures, practices and semantics of invention management in North-Rhine Westphalia (NRW, a former Western state) and Saxony (a former GDR state) to reflect on how academic institutions have been handling inventions along transforming socio-political contexts. Building on document analysis and qualitative interviews with research managers, we discuss ongoing differences in practices of invention management and the semantic framing of the societal value of inventions in NRW and Saxony, and discuss how this can be understood before the background of their ideological, political and economic separation until reunification in 1990. Joining the conceptual perspectives of path dependencies and sociotechnical imaginaries, we argue that two critical incidents in the history of these states (the reunification in 1990 and a legal change in 2002) allowed for wide-ranging institutional alignments, but also allowed path dependencies in practices and semantics of invention management to prevail.

“In the higher education sector there are not many aspects in which the GDR [German Democratic Republic] lived on and strangely enough…– I say it upfront – it is in this capitalistic part of higher education that the old GDR-system lives on.” (RM13)^1^

## Introduction

Expectations about universities have changed and broadened quite radically in recent decades, most prominently around the idea that knowledge production contributes to economic growth and societal wealth (e.g., Chesbrough 2003; Ulnicane 2015). In many countries around the world, science and research policies emphasize a notion of innovation that demands from universities a stronger focus on societal impact and technology transfer (Gibbons et al. 1994; Etzkowitz and Leydesdorff 1997; Felt and Glanz 2004; Geiger and Sá 2008; Dill and Van Vught 2010; Lundvall [1992] 2010; Block and Keller 2011).

In this context, the question of how inventions by scientists at universities can and should be managed to best serve societal needs has been widely discussed and has led to far-reaching institutional and legal changes. Most importantly – and often (at least discursively) taking the US Bayh-Dole Act of 1980 as a model – many European states have shifted from inventor ownership to institutional ownership of respective patents (Geuna and Rossi 2011). In the German science system, this required legal changes to abolish the so-called “professor’s privilege” according to which professors enjoyed full intellectual property rights (IPR) to their inventions. The quote above reminds us that the new institutional ownership of intellectual property rights resembles regulations during state-socialist times (like in the GDR up until 1990). As we will show in this paper, however, these similar institutional regulations were – and still are – embedded in quite different imaginaries of the role of science (and inventions in particular) for societal development, having interesting repercussions for how inventions are managed.

Understanding the entanglements between institutional and political transformations and lived practices is relevant today as IPR regulations in European universities are still quite diverse due to the heterogeneous “institutional, cultural and organizational landscape(s) surrounding academic knowledge transfer” (Geuna and Muscio 2009). While there is a general agreement that patent data are an insufficient metric for grasping the broader phenomenon of technology transfer (Wissenschaftsrat 2016), the quantity of patented inventions is still often used as a key indicator of innovation and research commercialization (Dornbusch and Neuhäusler 2015) because patent data are widely available and easy to compare, and other – or better – indicators are absent (Bornmann 2013). This absence is increasingly discussed within the field of Responsible Research and Innovation (RRI), which aims to institutionalise a new, and more responsible relationship between science and society (Owen et al. 2012). The challenge of such attempts, however, is that the meaning of societal impact depends on specific contexts and semantics that are involved in infrastructures and practices of invention management.

The major differences between East and West Germany illustrate this: even though the Eastern state Saxony (app. 4.05 million inhabitants) registers as much as 142 university patents per year, and the Western state North Rhine-Westphalia (NRW) (app. 18 million inhabitants) only registers 70 university patents per year, experts we interviewed for our study would agree that Saxon patents typically have less economic impact. Mere patent data thus are limited in their ability to reflect what scientific research can and should accomplish in and for society. To better understand the “complex relationships between institutions and their members” (Wickson and Forsberg 2015), we thus need to understand invention management as a situated practice of meaning-making within concrete socio-political contexts.

In this paper, we contribute to this through a comparative analysis of the infrastructures, practices and semantics of invention management in two German federal states. This comparison is interesting, since we can expect different path dependencies in East and West Germany because they were separated ideologically, politically and economically between 1949 and 1990. During this time, they developed divergent ideas about what constituted good science and what (and how) science can and should contribute to society. While these two scientific sub-systems were merged after reunification in 1990, a certain regional autonomy was preserved in the German federal governmental system. Specifically, we compare Saxony, a state in the East that was previously part of the German Democratic Republic (GDR), and NRW, a state in the West, and work out the different ideas that are implicit in how inventions were (and still are) managed in the two states. Further, we discuss the potential implications of these ideas for patenting policies today. We approach this comparison using a framework that combines the concepts of path dependencies and sociotechnical imaginaries.

We will argue that the German reunification with rather strong regional (state) autonomy allowed for wide-ranging institutional alignments, but also allowed existing path dependencies in practices and semantics of invention management to prevail, resulting in different ways in which legal changes were taken up in both states after 1990. Analyzing strategic and policy documents, as well as qualitative interviews with research managers in the two German states, we will show interesting path dependencies – especially in Saxony, one of the former socialist states in Eastern Germany. This opens up a space to reflect upon the imaginaries about the role of science (and inventions in particular) for societal development that our systems of invention management build on today.

Such differentiating analyses of how links between science and society develop in often discontinuous, ambivalent and uneven ways (Kleinman and Osley-Thomas 2014) are timely and relevant beyond the specific case of Germany, since the question of the public value of research and innovation is being renegotiated, for example, in the context of the policy framework RRI (cf. Hartley et al. 2016). It links to the heated debate about what role the state should take in innovation processes and to what degree it has a responsibility to be an “entrepreneurial state,” i.e., to act as the prime risk-taker in innovation management (Mazzucato 2013). Finally, our analysis will allow us to reflect upon the often implicit but quite normative ideas about who should benefit and who should bear the risks of the uncertain business of making use of inventions.

## Conceptualizing Continuity and Change in Technology Transfer

It is often pointed out that there has been a rapid change in how we conceptualize the role of science and technology in our societies, while, in contrast, there has been a remarkable continuity in academic practice. Especially the German science system has been described as slow to adapt to new expectations (Lange and Krücken 2011), and it has been said that “the discourse on university reform [was] not met at the level of organizational practices” (Krücken 2003a; cf. Kehm et al. 2010). Mainly based on observations from former Western German universities, this has been attributed to a strong preservation of Humboldtian ideals of unity of research and teaching, institutional barriers to the third mission of research commercialization (Krücken 2003b) and a tradition of recruiting managerial staff who lack business or legal expertise (Lange and Krücken 2011). Such accounts of a strong linear path dependency, however, have often focused on Western German states (mostly NRW), thereby silencing the institutional developments and experiences in former Eastern states before reunification in 1990 that later transfer policies could build on.

This absence is surprising, given that we have to assume different path dependencies, in the sense of the ways “by which institutions and beliefs derived in the past influence present choices” (North 2005). It has been shown that strong path dependencies have shaped the creation of technology transfer structures and processes in various countries (Dill and Van Vught 2010; Leišytė 2011), particularly in the spread and diffusion of technology transfer models in the US. Studies of institutional resistances to change in and among organizations have often perceived path dependence to be a driver of radical inertial forces (see, e.g. Powell 1991; Holm 1995). For example, Leišytė (2011) has shown how path dependence shapes the process of research commercialization in terms of the timing and the types of policy instruments that form the institutional framework conditions in the US and the Netherlands. Garud et al. (2002) have further indicated that path dependencies are crucial in the institutionalization process of innovations. It is thus important to understand what fosters or inhibits organizational change in technology transfer and how “critical junctures” (ibid.) (e.g., military conflicts, economic crises or other serious changes to the economic, political and social systems) (Hall and Taylor 1996) have the potential to break the path and create a new path.

In the context of the German reunification, institutional “critical ruptures” did not seem to correlate with equal ruptures in how meaning was attached to concrete everyday practices at academic institutions. To understand the relationship between institutional developments and the semantics of everyday practice, it thus seemed necessary to consider the specific historical ideas about the role of inventions in society. Following the historical institutionalist tradition, we thus also looked at routines and norms that are embedded in the organizational structure of political economy. Beyond the narrower institutionalist tradition, we looked at all “relatively persistent features of the historical landscape” as “central factors pushing historical development along a set of ‘paths’” (Hall and Taylor 1996). This approach seemed better equipped to explain change as based on the contextual features inherited from the past. Still, historical institutionalism emphasizes the rules or conventions promulgated by formal organization. In our attempt to integrate this perspective with other factors (such as ideas) that contribute to continuity and change in technology transfer, we find inspiration in the conceptual framework of sociotechnical imaginaries for two reasons:

First, it allows us to specify what kind of path dependency we look at: path dependencies in visions of science–society relationships. According to Sheila Jasanoff, institutions play a powerful role in this since “they control well-demarcated tracts of physical or virtual territory in which they exercise authority and implement the rules of the game” (Jasanoff 2015a). Because of this, institutions are important in stabilizing “sociotechnical imaginaries,” which are defined as “collectively held and performed visions of desirable futures” (Jasanoff 2015b). While being “temporally situated and culturally particular” and subject to change, such imaginaries have often been described as quite durable due to the stability of institutions, but also of social arrangements, identities, practices, experiences, visions and belief systems that give them value and meaning (ibid; cf. Jasanoff and Kim 2009). The concept of “sociotechnical imaginaries” thus implies the idea that societally and historically situated sociotechnical arrangements are coproduced by specific ways of meaning-making, however, in sometimes discontinuous and non-deterministic ways. In our specific study, it raises the question how the science–society relationship was reconfigured after reunification and how far institutional changes coincided with changing sociotechnical imaginaries (or not). We study the continuities and ruptures in this coproduction by looking at the historical development of infrastructures and practices of invention management at academic institutions in Western and in Eastern Germany.

Second, combining the concepts of path-dependency and sociotechnical imaginaries offers us conceptual breadth to consider institutional changes but also broader sociopolitical and cultural continuities and transformations. Specifically, the concept of sociotechnical imaginaries allows us to conceptually grasp whether and how imaginaries of invention management changed beyond the political units of nation states, in our particular case, along the discontinuous and partly abrupt transformation of the political units of states of Germany. As Sheila Jasanoff recently clarified, imaginaries can also “be articulated and propagated by other organized groups, such as corporations, social movements, and professional societies. … When a ‘vanguard vision’ … comes to be communally adopted … does it rise to the status of an imaginary” (Jasanoff 2015b). For the purposes of our paper, we thus speak of “imaginaries of invention management” defined as normative ideas about whether and how inventions (should) be managed to contribute to societal development in the best way. Imaginaries are multiple and can coexist “within a society in tension or in [a] productive dialectical relationship” (ibid.). This gives us a wide enough analytical angle to understand “both durability and change” of imaginaries and their “embedding, or rootedness” in infrastructures for and practices of invention management (Jasanoff 2015b; cf. Felt 2015).

In this paper, we describe imaginaries that are widely enough held to shape and guide practices of invention management at academic institutions in the two states of Germany we studied. In doing so, we consider that they have followed very different developmental paths of science–society relations between 1949 and 1990. Even if their scientific systems may have had similar institutional and cultural roots, we assume that they had embedded quite different ideas about what science, and inventions in particular, can and should contribute to society’s institutions, cultures, practices and identities. We propose that critical junctures in the development of science–society relations – notably the institutional changes after the German reunification in 1990 and a major change in the legal regulations for patenting at German Universities in 2002 (see below) – were experienced (and taken up) quite differently in relation to the specific imaginaries. We approach the question of continuity and change by looking at concrete infrastructures for and practices of invention management and the semantics of the science–society relationship they are coproduced by. We propose that these infrastructures, practices and semantics have taken different paths in the past 25 years in relation to the two critical junctures, but that they may now be converging into a shared imaginary of invention management.

## Paths of Technology Transfer in East and West Germany

Up until the 1990s, IP management was little institutionalized and a niche activity for academic institutions in the Western Federal Republic of Germany (FRG) (Krücken et al. 2009). Via the so-called professor’s privilege in the German Employee Invention Act (“Arbeitnehmererfindergesetz”) professors were free to register their patents themselves, to license them out to companies or, as was often the case, to assign their patents to a collaborating industry in return for project funds (Proff et al. 2012).

Contrary to this, invention management at universities in the Eastern GDR was well developed and embedded in the so-called innovator movement (German: *Neuererbewegung*).^2^ The state-led movement to support and improve productivity was increasingly legally regulated and reinforced beginning in the 1950s and resulted in a high political and economic status of inventors. These so-called innovators (*Neuerer*) suggested innovations in production processes at companies (the so-called “combines,” German: *Kombinate*), agricultural cooperatives, or higher education institutions. This was strongly regulated and institutionalized, following the idea that innovations could and should be centrally coordinated (Koblank 2012; Fritsch et al. 2015). At universities, so-called patent engineers had specialized education and patent departments that could be three to four times bigger in size compared to today’s technology transfer offices in Germany. Careful estimates suggest that institutional benefits were higher than the costs of such a system (Koblank 2012). Individual researchers got rewards for inventions, but the invention was state-owned.

After 1990, the Eastern states adopted the FRG legal framework for technology transfer, including the professor’s privilege. During the 1990s, invention management at universities gained political attention in the reunified Germany. Amongst others, targeted policy and funding measures were developed to improve competencies for managing and exploiting IP at universities (e.g., enlargement of legal departments and TTOs; BMBF 2000), and the routine privatization of intellectual property (IP) created in public universities (that was mostly patented by researchers individually or by collaborating industry) came under increasing scrutiny.

So after 2002, in the whole of Germany – like many other European countries at the time – the IP legal framework was changed as the professor’s privilege was abolished (Proff et al. 2012). Since then, public universities are entitled to the inventions of their staff and researchers are obliged to report any patentable inventions before publishing them. The university then assesses the invention’s patentability and economic potential, provides services and covers all costs involved in the patenting process. If the university decides to register the patent, it formally owns the patent, but researchers are entitled to 30% of the gross income,^3^ which is generally seen as a very generous solution for researchers. While providing an additional source of income for academic institutions, the legal change also got university administration under pressure to maximize rewards via licensing contracts or patent-mediated collaboration with industry, requiring “new infrastructures … to fulfil the new institutional task” (Krücken et al. 2007).

Further, the federal government started the “Exploitation Offensive” (“Verwertungsoffensive”) in 2002 and established so-called “Patent Exploitation Agencies” (“Patentverwertungsagenturen-PVA”). Today, these agencies operate as service agencies (mainly for universities) assessing the quality of inventions and their commercialization potential (Kulicke 2014). Since these changes, and following an international trend, the number of world-market-relevant patents doubled in the 15 years between 1997–2012 (from 185 to 370; BMBF 2015).

This movement towards institutionalizing the so-called third mission is often depicted as continuous and linear integration of IP management. Empirical studies, however, often do not distinguish between Eastern and Western states (Krücken et al. 2007; Krücken et al. 2013), making it difficult to see historically grown specificities. Although, on an institutional level, East and West Germany were aligned, statistics still suggest differences in patenting practices: e.g., university ownership of patents is still much more prevalent in the East than in the West (Ledebur et al. 2009). This – and the above-cited ongoing significant differences in numbers of university patents between different states – has led authors to call for studies of the differences between the states (cf. Schoen and Buenstorf 2013).

## Research Questions

This raises the question of why transfer activities (incl. invention management) continue to be so dissimilar. We approach this by asking three sub-questions: How did infrastructures for and practices of invention management change over time? How do research managers in the East and the West experience changes in the meaning of invention management at universities? And how far do different infrastructures, practices and semantics of invention management reflect different imaginaries about the role of inventions for societal development? In asking these questions we specifically consider continuities and changes linked to two critical junctures, reunification in 1990 and the legal changes in 2002.

## Empirical Settings, Material and Methods

To tackle these questions, we compare invention management in two German states: NRW and Saxony. Germany is a federal state that grants the 16 states (*Länder*) a great deal of autonomous decision-making. This leads to differences in the rationales for university organization and with regard to political commitment to technology transfer: NRW has had a strong political commitment to institutionalizing technology transfer since the 1970s but regularly questions whether this institutionalization actually translates into changes in practice (Lange and Krücken 2011). Less is known about Saxony. Statistics suggest rapid increases in R&D intensity in industry (BMBF 2014) but that the university system still depends on high investment from the public sector (ibid.).

NRW and Saxony are among the four most patent-active states in Germany: Saxony, the ex-GDR-state, is first and NRW, in the West, fourth (DPMA 2015), corresponding to an overall East–West divide, with the East contributing to overall university patenting above the national average (cf. Table 1). Before 2002, just two Eastern states, Saxony and Thuringia, were responsible for almost half of all patents filed by German universities. Even though the number of overall patent applications per inhabitant is higher for NRW, Saxony is strongest in university patenting with a figure more than double the one of NRW (DPMA 2015; Pasternack and Hechler 2007).

**Table 1:** Comparison of patents in NRW and Saxony. *Sources*: (BMBF 2015; DPMA 2015)

	Inhabitants	Patent applications / 100,000 inhabitants (2012)	University patents/year (2014)	GERD (2011)
NRW	17.84 million	38	70	2.02
Saxony	4.05 million	26	142	2.92

Triangulating different methods (Flick 2008), this study builds on an analysis of policy and strategic documents from research institutions, as well as qualitative interviews with research managers (employed at transfer-oriented universities and intermediary institutions that service universities) between January 2015 and November 2016. In NRW, the empirical work covers one technical university and one transfer-oriented academic research institution. In Saxony, it covers two universities (one technical university, one transfer-oriented comprehensive university). Since invention management in Saxony has a much longer history and is more strongly institutionalized, patenting practices and infrastructures are much better documented and represented in publicly available strategic and policy documents that – during GDR times – were also used for educating so-called “patent engineers” (professional invention managers). To balance the relative scarcity of respective documents in NRW, interviews play a more important role in our analysis of NRW. The analyzed documents were published by respective university departments (e.g., patenting strategies) but often include articles by institutions that provide patent management services for a broader range of academic research institutions in the respective states (e.g., by “Patent Exploitation Agencies”). The analysis focuses on imaginaries of the role of inventions and invention management for societal development, as reflected in the ways in which strategic aims are discursively embedded in these documents. It follows Prior (2003), who thinks of such documents as an independent genre that is meant to imagine and act upon the (research) system. In this understanding, policy documents are “situated products, rather than fixed and stable things in the world” and “collective (social) products” (Prior 2003).

To complement the document analysis, we conducted nine interviews with research managers in NRW, four in Saxony and one complementary and contextualizing interview with a research manager in Baden-Württemberg, who is widely regarded as an expert on the development of the patenting system in Germany. All interviewees were experienced in patenting practices (either within academic institutions or in servicing them) and the building of patenting infrastructures at German universities (e.g., IP management at universities, developing patenting strategies). Not all interview partners were employed at universities at the time – two had recently retired, and some had moved to other research institutions. Some interviewees had already been active patent managers before German unification, and almost all interviewees have either observed – or have actively shaped – how universities adapted to changes after the abolishment of the professor’s privilege in 2002. The questions asked about their biography in patent management, and about how they learned to cope with critical junctures. How they experienced these ruptures and controversies around them was particularly productive in making implicit assumptions about the use and meaning of invention management traceable. Interviews took approximately 90 minutes and were conducted face-to-face or on the phone. The analysis of the interviews considered that retrospective reflections do not reflect objective historical events. Avoiding the trap of the biographic illusion (Bourdieu 1990), the interviews are used to analyze how research managers in the present reconstruct historical continuities and junctures, thereby assigning meaning to their practices within a historical context.

This study followed a Grounded Theory approach (Glaser et al. 2005) in that the hypothesis developed out of a less focused study on research commercialization and was then narrowed down to more specific interests and theory building. The main hypothesis of this paper – that practices of invention management continued to follow different imaginaries regarding the role of inventions for societal development after reunification and legal changes in 2002 – was developed by building on a broader study on research commercialization^4^ and researcher statements about the highly bureaucratic invention management in NRW, which they saw as contrasting with the smoother IP management processes in Saxony. Our analysis of the data followed the Grounded Theory and a respective open coding process. The analysis was assisted by the NVIVO coding software.

The combination of document analysis and interviews offers a broad range of accounts of sociotechnical imaginaries in infrastructures and practices of invention management, with a particular emphasis on how institutional changes were taken up. Following such regulatory interventions and “how social actors and institutions respond when confronted by events that might disrupt order” is “a sure guide” to learning about patterns of meaning-making that underlie respective imaginaries (Jasanoff 2015b). In this sense, our research design allowed us to study whether the officially laid-out aims and goals of patent management institutions resonate with the rationales that govern actual practices.

## Changing Infrastructures and Practices of Invention Management

### Privatized Invention Management in NRW before 2002

Before 2002, patent management was a quite alien business to research managers at academic institutions in NRW. Indeed, during the 1990s most German universities had established technology transfer offices (TTOs) but even though they were acting as strongly proactive matchmakers between industry and university professors, the details of negotiating and managing inventions once collaborations were established remained outside of their realm. Patenting remained largely in the hands of professors, who could register their patents individually (assisted by a patent attorney), or in cooperation with industry. While in theory it was possible for professors to assign their inventions to the university, this option was rarely taken. One research manager stated that they only had to deal with a total of three inventions:

“between ’93 and 2002, we only had three patent documents in our drawer. Because inventions were handled by the professors back then. And the university did not see them. Those were three exceptional cases in which inventors registered it and left the whole [patenting] process to the university. …And now, we always have between 30 and 50 registrations per year.” (RM6)

Invention management was almost exclusively delegated to professors’ willingness and entrepreneurial skills. The legal change in 2002 was thus perceived as a huge challenge for the institution (e.g., for patent administration and negotiations over IP with industry) and the identity of academic researchers. It was met with enormous criticism by professors, who defended their right to their intellectual property as “academic freedom,” and the measure was even taken to court (RM13).

### State Involvement in Invention Management in Saxony before Reunification in 1990

Until 1990, all inventions at universities in Eastern Germany were state-owned and the management of inventions was thoroughly institutionalized, including reward systems, consciousness raising (the idea of solving societal problems by inventions) and socializing the young to value and crave inventions. The strong state involvement is still vividly remembered by a head of a technology transfer department as involving “orders to invent certain things,” as well as “bonuses, remunerations, some incentives to be inventive” (RM10).

The incentive structure at universities made sure that inventing paid off financially: Rewards of up to a one-month assistant’s salary were provided for every registered patent, regardless of future use or exploitation. Beyond that, universities allowed researchers to work on applications, which is seen in retrospect as morally and motivationally rewarding, as was described, for example, by a researcher who later chose a career as a patent engineer in the GDR era (cf. RM11). The now-retired patent engineer is described by his successor as not only extremely knowledgeable and smart, but as someone who “*really lived this patent thing with science. … It wasn’t a job for him that you do on the side, it was a passion”* (RM10). Patent management in the context of the innovator movement thus seems to have generated a self-motivation, transforming an institutionalized duty to invent into an activity that was both financially and personally rewarding.

The state also ensured that patenting was rewarding for academic institutions. Performance agreements stipulated that high numbers of registered patents could result in higher allocations. As a result, large technology transfer offices (TTOs) were established that handled the whole range of transfer activities (assessment, registering patents, exploitation, development). They are described as being similar to current TTOs, but much larger (in this specific case: eight professionals, as compared to currently two people) and with specialized professional categories (so-called “patent engineers”) (RM11). All this made it “easier to patent” (RM11) back then, since it required less outsourcing of tasks (e.g., to patent lawyers) and lower additional direct costs.

### Invention Management in Saxony after Reunification in 1990

Reunification brought major institutional reforms whose purpose was to align Eastern universities with Western standards of university organization. This also affected infrastructures and the legal basis of technology transfer: transfer departments were downsized, staff was reduced and professors were now entitled to own their inventions. Nevertheless, as a patent manager who had migrated from the West to the East only a few years after reunification explains, it remained somewhat self-evident that professors would deliver their inventions to the university, who then handled the registration:

“In the GDR, the invention was naturally owned by the university, i.e., the state. And professors were used to that. …[T]hey didn’t even become aware that they were the owners of their inventions. Rather, they continued to register their inventions at the university, just as they always did.” (RM13)

Others describe similar continuities, saying that “the established patent management system … was ultimately just continued” because “there were respective structures that could cope with the registration and support and – to a certain extent – also the exploitation” (RM14). The period between 1990 and 2002 is described as a state of legal exception that did not last long enough to reach the mind-sets of people. Transfer documents a few years after reunification still reflected continuity in the meaning of inventions, which were seen as enhancing the technological capacities of a society rather than as economic assets of academic institutions:

“The main scientific potentials also lead to the current need for automatisation of processes, machines and apparatuses… and respective partial solutions.” (TransferLetter 01.93)

The relative continuity of patenting practices is remembered as the result of the solid legal and tacit knowledge and skills of patent engineers who made sure to “have an efficient strategy to register patents and find partners for their exploitation” (RM10). Even though these skills are in retrospect often doubted or even seen as unfitting to the newer institutional conditions (RM11, RM13, RM14), the self-confidence with which the then-acting staff still made sense of their practices allows to conceive of the naturalness with which patenting practices continued.

### 2002 as Bombshell in Invention Management in NRW

In NRW, the abolishment of the professor’s privilege is discussed as a major turning point. Universities now shifted their focus to educating scientists about patenting, and searched for ways of integrating it into the identities of researchers. For example, a patent strategy envisaged a range of “sensitising measures,” such as workshops and information campaigns to “get the scientists to learn about their legal duty [to report inventions] and the [support services] of the technical university” (RM5, cf. Doc1). The time since 2002 is narrated as one of ongoing institutional learning and processes of change, for example, learning to work together with patent exploitation agencies, learning how to effectively decide on which patents will be filed (e.g., in university patent commissions), how to simplify procedures and how to negotiate with industry. Research managers in the West tend to emphasize the need to develop leaner management, since, as the head of a legal department explains:

“In the meantime, there are so many inventions because of the abolishment of the professor’s privilege, that the number became too high for the commission to deal with every single one… in essence, I am responsible for managing this, except for special cases in which the patent commission is consulted.” (RM6)

Meanwhile, the procedure had been boiled down to seeking the opinion of the inventor and matching it with the assessment by an exploitation agency and the strategic decision of the head of the legal department.

A bottom-line narrative is that patenting activities have started to stagnate (RM6, RM5.2) and that at this point in time, the primary aim is to maintain this level and avoid stark increases since patenting is as quite a costly investment that can “cause pain” if it gets too high (RM4).

### Invention Management in Saxony after 2002

In contrast, 2002 is not remembered as such a “bombshell” (RM10) in Saxony, but rather as “restor(ing) the old GDR conditions as a modern legal condition” (RM13). Managers could embed the practices that the new legal situation required into an existing and experienced community of practice. Reflecting on the success of the exploitation agency in Saxony (SPVA) shortly after the legal change, the head of the SPVA summarizes that the “contacts to industry and networks of GWT [i.e. the society for knowledge and technology transfer] are a basic precondition for the success of SPVA” (TransferLetter 01.03). These contacts and networks are seen – at least in part – as having been carried over from the history of patent management in the GDR era in technical universities and full-scope universities alike (RM10, RM14). Despite a generational change since then, institutional capacities that – in 2002 – were informed by almost four decades of experience seem to have been continued on through to today: the SPVA, for example, could build on a private transfer company that had already been established at the end of the 1990s and whose staff had been educated in the GDR and had long-established networks. The dense net of tailored services for technology transfer that exists today is frequently traced back to the historically strong support from both the management of academic institutions and the state government (e.g., RM11). Managing directors (“Chancellor”) at universities are often described as being strongly committed and generous with financial support for proactive invention management:

“Already in the 1990s we had a Chancellor who strongly supported the patent management system, and who also took care that the university had significant financial resources. You have already… mentioned the high numbers of patents… and you are right, we are frontrunners in Germany. And this is because the university has invested a lot. That is of course a strategic decision.” (RM13)

The same manager continues to explain that even academic management (“Rector”) still strongly identifies with the idea of being a “transfer university” (RM13). Resonating with such narrations, technology transfer activities are strongly represented, e.g., in written documents (such as a journal called “TransferLetters,” an information system for industry [German: “Dresdner Transferbriefe”]), intermediary services (customer service support, the negotiation and design of contracts, staff management and administration of technology transfer), special course offerings for scientists and teaching programs (TransferLetter 2.16). In addition to these institutional commitments, the commitment of the state government was considered to be stronger than in some Western states – e.g., through complementing federal budgets for patenting programs (RM13). However, this was also considered to be less influential than the commitment of managers in the academic institutions.

## Divergence in the Semantics of Invention Management: Patenting as an End in Itself vs. Patenting as a Means to an End

Research managers both in Saxony and NRW often refer to two opposing sociotechnical imaginaries of invention management, conveying two different semantics of the role of science (and inventions in particular) for societal development: the first sees patenting as means to very broad and longer-term societal aims. Since these aims often go beyond the range of current vision, patenting often appears as *an end in itself* in concrete practices of invention management. In contrast, the second imaginary implies the idea that an invention is only worth to be patented if one can already foresee not only potential applications but very concrete opportunities for commercialization. It directs invention management towards short-term, quite narrow, and often economic ends, which is why we refer to it with the shorthand *patenting as a means to an end*.

The GDR patenting system followed the first imaginary: patented inventions (regardless of future use) and a mind-set of valuing inventions were seen as benefiting socialist society in the long run. As an influential patenting handbook for scientists from 1984 explains, the GDR system was explicitly positioned to oppose capitalist legal frameworks that allowed private exploitation of inventions and was designed for socialist society to secure its “right to economic exploitation of inventions that are created in businesses (“Betrieben”) and institutions (“Einrichtungen”) and to guarantee inventors their due moral and material rewards” (Doc3). This was operationalized in academic institutions as the aim to produce high amounts of inventions. Even today, as a new legal framework is in place, the semantics of *patenting as an end in itself* are described as persisting, both in the practices of invention management and in the mind-set of acting staff. Especially the older generation of patent managers defends this against rather selective patenting (which is the norm in many Western universities):

“…if you do not have a broad patent portfolio, you have failed to position the issue of transfer in the university public in general. Then it is an issue that is exotic and rates a poor second.” (RM13)

The definition of what is worth publishing is still defined quite broadly, encompassing “unfinished, immature research results” (Doc2). This approach obviously requires extensive resources that, so far, seem to be quite generously provided by both university management and the state government (RM11, RM13, RM14). However, as one research manager suggests, this broad support may be shifting towards more focused support:

“I think that we are in a period of [change] right now. There is political pressure – you can sense it in the interest of members of parliament, Ministries – towards doing more serious patent exploitation, to see patents as a means to an end at this point… particularly in Eastern Germany probably: the money [that is put into the system] must be reduced at some point and the institutions prepare for that, and the states prepare for that, because the budget will be smaller and the competition between the recipients… becomes stronger…” (RM14)

This suggests that in Saxony, we may be observing a shift from a rationale of *patenting as an end in itself* towards *patenting as a means to an end*, in which patents are rather defined as assets whose value is defined by potential and actual exploitation. Discursively, this shift can already be traced to the period shortly after the abolishment of the professor’s privilege. At that time, the core interest expressed in transfer documents became focused on the overall technological capacity-building for the economic development of the region (TransferLetter 01.03). In 2006, patents and innovations were regarded the “unique selling points,” securing the “right to prohibit application by competitors” and “securing one’s own position on the market” (TransferLetter 02.06). Even if, on the level of written documents, semantic convergence with the West seems to have been relatively quick, research managers doubt that their practices or “key figures already reflect those aims” (RM10). In contrast, the rationale of *patenting as a means to an end* is much more firmly established in NRW, both semantically and in practice, with patents being narrated as contribution to the quality of science, as generating income for universities (via licenses or third party funds), as contributing to regional development or as key to establishing collaborations with industry. Patenting is still experienced as a rather new institutional task, and there seems to be a greater need for legitimization within the university public. The need to patent is often embedded in a rhetoric of enlarging opportunities for doing research, implicitly defining research as core academic activity and the so-called third mission as only catering to – and optimizing conditions for – this core. This same argument is also described as having been decisive in settling a legal debate over whether it was legitimate to confine property claims to inventions by professors in 2002. As a patent manager who was involved in the debates explains:

“Of course, it [the legislature] intervenes in the property positions of professors and in the freedom of research. But the motivation of the legislature, to open higher education institutions a new source of income, is a legitimate motive.” (RM13)

Particularly in the first few years after 2002, this argument was quite popular amongst research managers in NRW. And even though, in the meantime, most seem to agree that patenting does not increase university income directly and that the “business of inventions is a loss-making business,” the argument has now shifted to the more indirect ways that science profits from patenting – for example, that “it is good for the reputation and acquiring third party funds” (RM6). This is often linked to an understanding that research managers have a responsibility to contribute to regional development:

“… you have to consider the market, but of course you can steer that in a way. You try… to build value chains in the region, in the state or in the federal state. …you also have a certain responsibility for the economic and social development. That sounds a little pretentious, but… You prefer to have a contract with a firm in the region or in the state over a firm outside of Europe.” (RM8)

Investments are constantly matched to expected returns, and strategic aims of the university. The emphasis here is on the narrower economic ends of “providing core offers to industry” (RM9) and of contributing to the competitiveness of individual universities.

In contrast, patenting in Saxony still does not require such legitimation. The value of patenting is rather taken for granted despite an agreement that patents never operate in the black in terms of direct financial returns (RM11). However, some insinuated that, in the recent past, the idea that inventions should be of immediate economic value is gaining ground. A patent expert in the West observes that the narrower economic (“betriebswirtschaftliche”) motives may slowly become exchanged for broader national economic (“volkswirtschaftliche”) motives. In the German language, “betriebswirtschaftlich” relates to narrower institutional “business” economy of a university (semantically similar to *patenting as a means to an end*), while “volkswirtschaftlich” refers to patents as indirect means of supporting the “national” economy (semantically similar to *patenting as an end in itself*) (RM12):

“Until 2007, everything moved towards the business-economy perspective… [T]hat was the dream… for which American universities had set the example with their astronomic licensing incomes. … [Then] the phrasing changed from licence income to impact on society… along the lines of: we do not just collect money, but we have an influence on society.” (RM12)

The interviewee links this semantic change to a terminological shift towards “innovation”, and unmet expectations about the narrower economic value of patents for universities (e.g., in the context of biotechnology around 2000). In his interpretation, the absence of political legitimation for the support of patenting at universities was filled with “a new political argumentation,” emphasizing the “impact on society” and “the national economy character” (RM12). Interestingly, this is reminiscent of the semantics of *patenting as an end in itself* that we know from the Eastern context in Saxony. Such accounts of change suggest that we might currently be witnessing a moment of semantic turbulence that future studies will need to observe and reflect upon.

## Discussion

Our comparison of the semantic structures in NRW and Saxony shows that different systems of invention management convey different normative ideas about who can and should benefit from patenting inventions (society, academic institutions, individual researchers), and different imaginaries about the responsibilities of the actors involved:

*(1) Different forms of invention management correspond to different imaginaries about the role of researchers and the meaning of academic freedom.* The strong emphasis on individual agency in NRW appears as semantically anchored in a particular idea about academic freedom: one that includes researchers’ sovereignty over their inventions and the entrepreneurial freedom to exploit them. How this conflicted with the meaning of academic freedom in ex-GDR states like Saxony after 1990 may be exemplified by how a patent manager in Saxony recalls the controversy around the abolishment of the professor’s privilege:

“When the law was reformed… the negative right to publish was included: the right of professors to let their inventions vanish in their drawers. No one in Germany had talked about that before, nobody! And with this law it became a completely new institution, from my perspective a completely surreal and redundant institution. But this was when the Justice Department realised: ‘Oh my god, there is this freedom of research and teaching and we have to consider that in the law!’ A professor, if he (sic!) is lucky to invent something, and then keeps it secret! That is completely remote from everyday life [German: ‘lebensfremd’]… in the East, the professors didn’t even know what they were talking about!” (RM13)

Expressions like “surreal,” “redundant” and “lebensfremd” show how strongly this Western idea of academic freedom was in tension with what was considered to be the professional duty of academics in the GDR. From a perspective that puts societal benefits first, it seemed inconceivable to hide inventions under the pretext of individual academic freedom. This corresponds with the more general collectivist mindset in GDR’s state socialism. From such a perspective, a “negative freedom” to publish would absolve scientists from the responsibility to contribute to societal development. Such an attitude can only make sense within a semantic structure that puts individual entrepreneurial freedom first, which is anchored in the idea that this kind of freedom is a precondition for a thriving economy.

*(2) There are implicit assumptions about the role of institutions and the question of who should assume the (economic) risk of invention management.* In Saxony, there was a robust link between the idea that every invention was beneficial to building a socialist society and the presence of a strong institutional hand managing inventions. This imagination about institutional responsibility goes beyond a mere division of tasks between researcher (to invent) and institution (to manage). It implies an idea about who should cope with the uncertainties of making use of inventions and with the risk that an invention might have high management costs without ever paying off in terms of economic or societal benefit. Interview partners in Saxony tended to highlight the state’s support of universities, start-ups and companies as “giving more security in carrying out the tasks” (RM10) and the emphasis on the state as risk-taker (RM11). One patent manager explained how this is an advantage in negotiations with industry: having a public institution with a high level of expertise gives “better leverage to the university” (RM10) and ensures that inventors trust universities to be able to manage their inventions.

Linked to this division of work, academic institutions saw themselves as switch points (TransferLetter 01.93) or intermediaries between science and the uncertain business of applying inventions (TransferLetter 04.09) in Saxony. In contrast, in NRW, researchers themselves are presumed to be the main nodes in the networks with industry partners, and it is also the scientists (and science) who are framed as the main benefactors, e.g., helping researchers to acquire “additional knowledge to position their research” within the international community (RM8), “to account for excellence and know-how” (RM2) and to acquire (more) industry funding (RM8). This difference helps explain why, in NRW, making researchers less risk-averse is seen as one of the most challenging tasks for patent managers today, while in Saxony, this debate is rather remote. In Saxony, the risks of invention management are traditionally handled by institutions, since the brokering between science and society is seen as an integral part of institutionalized services (e.g., of exploitation offices; RM10). As one research manager explains, today’s challenge is often seen in finding new, institutionalized forms of collective risk-taking, e.g., in that patent exploitation agencies operate “in an environment in which they strive for cooperation, to cushion the risks amongst each other and support each other” (RM10). The main challenge in Saxony is seen as learning to master an intermediary position, while in NRW it is about making researchers acquainted with market logics and about adding a task to the performance portfolio of excellent researchers.

## Conclusion: Persisting Path Dependencies along Institutional Convergence

In this paper we suggest, that while the invention management infrastructures in Eastern and Western Germany were structurally aligned after reunification, path dependencies persist on the level of practices and the semantics of invention management. Taking the states of NRW in the former West and Saxony in the former East as examples, we have discussed these path dependencies in terms of the different ways in which critical incidents – reunification in 1990 and the abolishment of the professor’s privilege in 2002 – were narratively framed and taken up. We have argued that they can be linked to different sociotechnical imaginaries that guide invention management. Our comparative analysis allows to understand that the movement towards more institutionalized invention management is – at least in the German context – less linear than is often assumed. It shows how research managers handled new legal conditions and societal expectations from their historically specific standpoints. By focusing on a comparison of the sub-national level, our research contributes to ongoing scientific debates over *path dependencies and societal imaginaries on the regional level* (e.g., Pfotenhauer and Jasanoff 2017). Conceptually, we broaden the debate about path dependencies by including an analysis of what we call semantic path dependencies.

Specifically, we show how invention management in the East still follows a semantics of *patenting as an end in itself* (or as a means to very broad societal aims) that can be semantically traced back to GDR semantics in which inventions helped building a socialist society. In Saxony, invention management is still to a great extent defined as institutional responsibility. In contrast, the West follows a semantics of patenting as a means to rather narrow economic ends (for the research institution or for the region). This implies quite *different ideas about what role inventions and invention management play for societal development*: in the West, scientists are still conceptualized as core actors in invention management.^5^ The managerial services by institutions are seen as primarily supporting and securing the academic and entrepreneurial freedom of the scientists.

This different meaning-making is reflected in persistent differences in patenting practices such as the very broad criteria for patenting an invention in Saxony vs. the narrower economic criteria for patenting in NRW. This suggests that invention management, and the so-called third mission in general, is still seen as an inherent part of the academic institutions in the East. In contrast, our empirical material – as well as other studies of Western universities – draw quite a different picture of how academic institutions engage with increased societal expectations: linkages to society are rather depicted as private activities (Krücken et al. 2009) and aims to strengthen technology transfer have been described as lacking required support by relevant actors (Lange and Krücken 2011). This supports the suggestion by Schoen and Buensdorf (2013) that differences between German states are important for explaining the strong and ongoing differences in patenting quantity.

These imaginaries also imply quite normative ideas about who should take the risks. The relatively generous practices of patenting many inventions (even those with little exploitation potential) in Saxony suggest that it is still public institutions who are seen as responsible to invest in the uncertain business invention management. Involved risks are accepted as part of the game and the aim is to share risks amongst public institutions. Patenting in this semantic framework is best understood as a system that cultivates a reflection on potential societal relevance. In contrast, the aim in NRW seems to be to minimize risk: the quite narrow patenting criteria (high economic potential) implicitly reject the idea that the institutions should bear the greatest share of the risk of invention management. Inventions whose application are more uncertain are re-assigned to the individual researchers who then can decide whether they want to take the risk or not. In that sense, the individual researchers in NRW are called upon to be entrepreneurs – and to assess on their own whether an invention has economic potential. This is much less the case in Saxony, where the path to actual commercialization is paved with more intermediary structures that de-couple practices of inventing from the logics of commercialization. It will be of interest for future studies to consider how different intermediary structures have implications for how research cultures get entangled with the codes and practices of the commercial world (cf. Kleinman and Vallas 2001).

Our study indicates that we might today be observing a semantic shift as a result of *another critical incident: that of the economic crisis and, linked to it, unmet expectations about the economic potential of patenting at universities*. As Elizabeth Popp Berman has stated regarding the US context, “When the economy is stagnating and university budgets are being cut, it may make sense, strategically, for universities to declare themselves economic engines” (Popp Berman 2012). Our interview partners were nonetheless inconclusive about where a new semantic shift may be headed. A Western patent manager observed a semantic shift towards *patenting as an end in itself* while an Eastern patent manager observed a shift towards *patenting as a means to an end*. This suggests that semantic turbulences may lie ahead. How far they will play out in shifting practices and infrastructures of invention management in the future – and whether the framing of these shifts will align or again be linked to socio-historical differences – is an interesting topic for further research.

Finally, the argument of this paper, that the meaning of patenting is quite different depending on the institutional, practical and semantic contexts of its implementation, implies that *patent data reflect the societal impact or responsibility of researchers quite differently, depending on research field or institution*. A recent contribution to the debate over the German core dataset for research (“Kerndatensatz Forschung”), for example, states that patent data alone are not enough for determining innovation activities: transfer as a more encompassing phenomenon is treated here as an (as yet) “hardly describable term” in the context of innovation, and it is becoming important to all actors in the science system (Wissenschaftsrat 2016). The arguments of this paper suggest that, as indicators continue to be developed, it will be important to reflect on more tacit semantic and practice differences in how inventions are managed in their specific contexts. As our interview partners indicated, the high numbers of university inventions tell little about actual applications. Counter-intuitively to the much higher patenting rates in the East, the share of actually applied inventions can differ as strongly as 5% in an Eastern university to approx. 30% in Western ones (RM14). Further work on developing such indicators (e.g., third party funds, licensing incomes) should keep in mind that such key figures always express “crystallised” ideas of what innovations can and should achieve for a society (cf. Godin 2004). If patent numbers continue to gain influence in the measurement of research performance, we will require further research to understand the potentially different repercussions such indicators could have for practices of invention management in contexts that embrace different ideas about the societal role of inventions.

## List of Cited Sources for Document Analysis:

**Table:**

Transfer Letters (German: “Dresdner Transferbrief”; http://nbn-resolving.de/urn:nbn:de:bsz:14-qucosa-102900)	
TransferLetter 2.16	Thema: Leuchtturm Biotechnologie | DresdnerTransferbrief 2016, Volume 24, Issue 2
TransferLetter 01.14	Innovation am Bau | DresdnerTransferbrief 2014, Volume 21, Issue 1
TransferLetter 04.09	Thema dieser Ausgabe: Medizintechnik – Kompetenzen & Innovationen für die Zukunft | DresdnerTransferbrief 2009, Volume 17, Issue 4
TransferLetter 2.06	Thema dieser Ausgabe: Gewerbliche Schutzrechte | DresdnerTransferbrief 2006, Volume 14, Issue 2
TransferLetter 1.03	Thema dieser Ausgabe: Informations- und Kommunikationstechnik | DresdnerTransferbrief 2003, Volume 11, Issue 1
TransferLetter 01.93	cit. TransferLetter 01.14
Other documents designed to guide and educate invention managers	
Doc1	Patentstrategie, Technische Universität Dortmund (01.07.2016)
Doc2	Universität Leipzig: Erfindungen und Patente (https://www.zv.uni-leipzig.de/forschung/wissens-und-technologietransfer/erfindungen-und-patente.html; 28.06.2016)
Doc3	Matschiner B. (1988 [1984]) Patentfibel für Chemiker, Berlin: VEB Deutscher Verlag für Wissenschaften.

## Footnotes

1 “RM” is an abbreviation for “Research Managers” who we interviewed for this study.

2 In the absence of a better alternative, the German term “Neuerer” is here translated as “innovator.” We want to note, however, that the meaning of the term “innovation” today carries different meanings: particularly in research policy contexts, it is often narrowed to mean the mere commercialization of technological inventions (Godin 2008). As will become clear below, the meaning of being a “Neuerer” did not narrowly describe someone who simply produced inventions with economic value, rather it was related to an understanding that inventions were a means to achieving broader societal aims in the context of the GDR.

3 “Gross income” in this context means that researchers are entitled to 30% of the income a university makes from a patent, before subtracting the costs that the universities had for managing and exploiting the patent. This is often criticized by patent managers as making patent exploitation unprofitable for the university.

4 The empirical work of this paper was part of the research project “University-industry collaboration in high-tech fields in the U.S. and Europe” (Project leader: Liudvika Leišytė); https://www.zhb.tu-dortmund.de/zhb/hdhf/de/forschung/projekte/uic/index.html (29.01.2018).

5 We do not want to imply that the role of researchers in the “West” has not changed. Many have argued that the expectations involved in making an academic career have changed (Müller 2012; Fochler et al. 2016) and that - against the background of new public management reforms, changing performance expectations and temporary contract cultures – we can observe shifts in identities and forms of subjectification (Leišytė 2015; Sigl 2016).

## References

[CR1] Block Fred, Keller Matthew R (2011). State of Innovation.

[CR2] BMBF - Bundesministerium für Bildung und Forschung. 2000. Bundesbericht Forschung 2000.

[CR3] BMBF - Bundesministerium für Bildung und Forschung. 2014. Bundesbericht Forschung und Innovation 2014.

[CR4] BMBF - Bundesministerium für Bildung und Forschung. 2015. Bildung und Forschung in Zahlen 2015.

[CR5] Bornmann Lutz (2013). What Is Societal Impact of Research and How Can It Be Assessed? A Literature Survey. Journal of the American Society for Information Science and Technology.

[CR6] Bourdieu Pierre (1990). Die biographische Illusion. BIOS Zeitschrift für Biographieforschung und Oral History.

[CR7] Chesbrough Henry W (2003). Open Innovation: The New Imperative for Creating and Profiting from Technology.

[CR8] Dill D, Van Vught FA (2010). National Innovation and the Academic Research Enterprise.

[CR9] Dornbusch, Friedrich, and Peter Neuhäusler. 2015. Academic Patents in Germany. In *Studien zum deutschen Innovationssystem Nr. 06-2015*, ed. Expertenkommission Forschung und Innovation (EFI): Fraunhofer Institute for Systems and Innovation Research.

[CR10] DPMA – Deutsches Patent- und Markenamt. 2015. Jahresbericht 2014: DPMA.

[CR11] Etzkowitz, Henry, and Loet Leydesdorff. 1997. Universities in the global knowledge economy. In *Universities and the global knowledge economy. A triple helix of university - industry - government relations*, eds. Henry Etzkowitz, and Loet Leydesdorff. London: Pinter.

[CR12] Felt, Ulrike. 2015. Keeping Technologies Out: Sociotechnical Imaginaries and the Formation of Austria’s Technopolitical Identity. In *Dreamscapes of Modernity*, eds. Sheila Jasanoff, and Sang-Hyun Kim, 103-125. Chicago & London: Chicago University Press.

[CR13] Felt, Ulrike, and Michaela Glanz. 2004. University Autonomy in Europe: Shifting Paradigms in University Research? In *Managing University Autonomy. Shifting Paradigms in University Research. Proceedings of the Seminar of the Magna Charta Observatory, 15 September 2003*, ed. Magna Charta Observatory, 15–99. Bologna: Bononia University Press.

[CR14] Flick Uwe (2008). Triangulation.

[CR15] Fochler Maximilian, Felt Ulrike, Müller Ruth (2016). Unsustainable Growth, Hyper-Competition, and Worth in Life Science Research: Narrowing Evaluative Repertoires in Doctoral and Postdoctoral Scientists’ Work and Lives. Minerva.

[CR16] Fritsch Michael, Sorgner Alina, Wyrwich Michael (2015). Die Entwicklung der Wirtschaft in Ostdeutschland nach der Wiedervereinigung. *Gesellschaft, Wirtschaft*. Politik (GWP).

[CR17] Garud Raghu, Jain Sanjay, Kumaraswamy Arun (2002). Institutional Entrepeneurship in the Sponsorship of Common Technological Standards: The Case of Sun Microsystems and Java. Academy of Management Journal.

[CR18] Geiger Roger L, Sá Creso (2008). Tapping the Riches of Science. Universities and the Promise of Economic Growth.

[CR19] Geuna Aldo, Muscio Alessandro (2009). The Governance of University Knowledge Transfer: A Critical Review of the Literature. Minerva.

[CR20] Geuna Aldo, Rossi Federica (2011). Changes to university IPR regulations in Europe and the impact on academic patenting. Research Policy.

[CR21] Gibbons Michael, Limoges Camille, Nowotny Helga, Schwartzman Simon, Scott Peter, Trow Martin (1994). The New Production
of Knowledge: The
Dynamics of Science and Research in Contemporary Societies.

[CR22] Glaser, Barney G., and Anselm L. Strauss. [1998] 2005. *Grounded Theory: Strategien qualitativer Forschung (Original Title: The Discovery of Grounded Theory)*. 2 Aufl. Bern: Huber.

[CR23] Godin, Benoit. 2004. The Who, What, Why and How of S&T Measurement. *Project on the Intellectual History of Innovation* Working Paper No. 26.

[CR24] Godin, Benoit. 2008. Innovation: The History of a Category. *Project on the Intellectual History of Innovation* Working Paper No. 1.

[CR25] Hall, Peter A., and Rosemary C.R. Taylor. 1996. Political Science and the Three New Institutionalisms. *Political Studies* XLIV: 936–957.

[CR26] Hartley, Sarah, Warren Pearce, and Alasdair Taylor. 2016. Against the tide of depoliticisation: The politics of research governance. *Policy & Politics.*

[CR27] Holm P (1995). The Dynamics of Institutionalization: Transformation Processes in Norwegian Fisheries. Administrative Science Quarterly.

[CR29] Jasanoff, Sheila. 2015a. Imagined and Invented Worlds. In *Dreamscapes of Modernity*, eds. Sheila Jasanoff, and Sang-Hyun Kim, 321–341. Chicago & London: Chicago University Press.

[CR28] Jasanoff, Sheila. 2015b. Future Imperfect: Science, Technology, and the Imaginations of Modernity. In *Dreamscapes of Modernity*, eds. Sheila Jasanoff, and Sang-Hyun Kim, 1–33. Chicago & London: Chicago University Press.

[CR30] Jasanoff Sheila, Kim Sang-Hyun (2009). Containing the Atom: Sociotechnical Imaginaries and Nuclear Power in the United States and South Korea. Minerva.

[CR31] Kehm BM, Michelsen S, Vabø A (2010). Towards the two-cycle degree structure: Bologna, reform and path dependency in German and Norwegian universities. Higher Education Policy.

[CR32] Kleinman Daniel Lee, Osley-Thomas Robert (2014). Uneven Commercialization: Contradiction and Conflict in the Identity and Practices of American Universities. Minerva.

[CR33] Kleinman Daniel Lee, Vallas Stephen P (2001). Science, Capitalism, and the Rise of the ‘Knowledge Worker’: The Changing Structure of Knowledge Production in the United States. Theory and Society.

[CR34] Koblank Peter (2012). Das Neuererwesen der DDR.

[CR35] Krücken Georg, Blümel Albrecht, Kloke Katharina (2013). The Managerial Turn in Higher Education? On the Interplay of Organizational and Occupational Change in German Academia. Minerva.

[CR36] Krücken Georg (2003). Learning the ‘New, New Thing’: On the role of path dependency in university structures. Higher Education.

[CR37] Krücken Georg (2003). Mission impossible? Institutional barriers to the diffusion of the “third academic mission” at German universities. International Journal of Technology Management.

[CR38] Krücken Georg, Meier Frank, Müller Andre (2007). Information, cooperation, and the blurring of boundaries – technology transfer in German and American discourses. Higher Education.

[CR39] Krücken Georg, Meier Frank, Müller Andre (2009). Linkages to the civil society as ‘leisure time activities’? Experiences at a German university. Science and Public Policy.

[CR40] Kulicke, Marianne. 2014. Working paper from the scientific research supporting the “EXIST – University-based start-up programmes”. Karlsruhe: Fraunhofer-ISI.

[CR41] Lange, Stefan, and Georg Krücken. 2011. German Universities in the New Knowledge Economy. Current Changes in Research Conditions and University-Industry Relations. In *Knowledge Matters. The Public Mission of the Research University*, eds. Diana Rhoten, and Craig Calhoun, 342–376. New York: Columbia Press.

[CR42] Ledebur, Sidonia von, Guido Buenstorf, and Martin Hummel. 2009. University Patenting in Germany before and after 2002: What Role Did the Professors’ Privilege Play? *Jena Economic Research Papers* (068).

[CR43] Leišytė Liudvika (2011). University commercialization policies and their implementation in the Netherlands and the United States. Science and Public Policy.

[CR44] Leišytė, Liudvika. 2015. Changing academic identities in the context of a managerial university – bridging the duality between professions and organizations. Evidence from the U.S. and Europe. In *The relevance of academic work in comparative perspective*, eds. William K. Cummings, and Ulrich Teichler, 59–73. Dordrecht: Springer International Publishing.

[CR45] Lundvall, B. A. [1992] 2010. Post Script: Innovation System Research – Where it Came from and Where it Might go. In *National Systems of Innovation: Towards a Theory of Innovation and Interactive Learning*, ed. B. A. Lundvall. London: Pinter Publishers.

[CR46] Mazzucato Mariana (2013). The Entrepreneurial State. Debunking Public vs. Private Sector Myths.

[CR47] Müller, Ruth. 2012. On becoming a ‘Distinguished’ Scientist. Individuality and Collectivity in Postdoctoral Life Scientists’ Narratives about Living and Working in Academic Sciences. University of Vienna, Vienna.

[CR48] North Douglass C (2005). Understanding the Process of Economic Change.

[CR49] Owen Richard, Macnaghten Phil M, Stilgoe Jack (2012). Responsible Research and Innovation: from Science in Society to Science for Society, with Society. Science and Public Policy.

[CR50] Pasternack, Peer, and Daniel Hechler. 2007. Forschungslandkarte Ostdeutschland. In *die hochschule. journal für wissenschaft und bildung*, ed. Institut für Hochschulforschung (HoF).

[CR51] Pfotenhauer Sebastian M, Jasanoff Sheila (2017). Panacea or diagnosis? Imaginaries of innovation and the ‘MIT model’ in three political cultures. Social Studies of Science.

[CR52] Popp Berman, Elizabeth. 2012. Economic Engines: At What Cost? *Inside Higher Education*.

[CR53] Powell WW, Powell WW, DiMaggio PJ (1991). Expanding the scope of Institutional Analysis. The New Institutionalism in Organizational Analysis.

[CR54] Prior Lindsay (2003). Using documents in social research.

[CR55] von Proff S, Buenstorf Guido, Hummel Martin (2012). University Patenting in Germany before and after 2002: What Role Did the Professors’ Privilege Play?. Industry and Innovation.

[CR56] Schoen Anja, Buenstorf Guido (2013). When Do Universities Own Their Patents? An Explorative Study of Patent Characteristics and Organizational Determinants in Germany. Industry and Innovation.

[CR57] Sigl Lisa (2016). On the Tacit Governance of Research by Uncertainty: How Early Stage Researchers Contribute to the Governance of Life Science Research. Science, Technology, & Human Values.

[CR58] Ulnicane Inga (2015). Broadening Aims and Building Support in Science, Technology and Innovation Policy: The Case of the European Research Area. Journal of Contemporary European Research.

[CR59] Wickson Fern, Forsberg Ellen-Marie (2015). Standardising Responsibility? The Significance of Interstitial Spaces. Sci Eng Ethics.

[CR60] Wissenschaftsrat. 2016. Empfehlungen zur Spezifikation des Kerndatensatz Forschung (Drs. 5066–16).

